# Mobile Apps and Wearable Devices for Cardiovascular Health: Narrative Review

**DOI:** 10.2196/65782

**Published:** 2025-04-04

**Authors:** Gauri Kumari Chauhan, Patrick Vavken, Christine Jacob

**Affiliations:** 1 FHNW - University of Applied Sciences Northwestern Switzerland, Bahnhofstrasse 6, Windisch, 5210, Switzerland, 41 562027464; 2 Vavken Health Lab, Zürich, Switzerland; 3 ETH, Zürich, Switzerland; 4 University of St. Gallen, St. Gallen, Switzerland

**Keywords:** eHealth, mobile health, mHealth, digital health, technology assessment, technology adoption, technology implementation, cardiovascular diseases, cardiovascular health, Germany, Austria, Switzerland, wearables, apps, smartphones, Swiss Apple App, Google Play Store, reviews, morbidity, mortality, well-being, care management, health technologies

## Abstract

**Background:**

Cardiovascular diseases (CVDs) continue to be the leading cause of global morbidity and mortality. Aiming to reduce the risk of CVD development and better manage them, an increasing number of individuals are adopting mobile health (mHealth) apps and wearable devices (wearables). These technologies provide critical insights into heart health and fitness, supporting users to monitor their lifestyle behaviors and adhere to preventative medication.

**Objective:**

In this review, we aimed to investigate the current state of mHealth apps and wearables designed for cardiovascular health, with a specific focus on the DACH region (Germany, Austria, and Switzerland). We assessed the benefits these technologies provide to clinicians and patients, particularly in addressing unmet needs like sex-specific symptoms, while also examining their potential integration into the broader health care ecosystem.

**Methods:**

To identify heart health apps, a keyword search was performed on both the Swiss Apple App Store and Google Play Stores. A separate search was performed on Google to identify heart health wearables. The identified apps and wearables were evaluated using the foundational and contextual criteria of the sociotechnical framework for assessing patient-facing eHealth tools.

**Results:**

After filtering out apps and wearables that did not meet our inclusion criteria, 20 apps and 22 wearables were included in the review. While all the apps were available in the DACH region, only 30% (6/20) were specifically designed for these countries. Only 25% (5/20) of the apps included sex-specific information; 40% (8/20) provided information from evidence-based research, 35% (7/20) provided general health information without academic and clinical references, and 25% (5/20) did not include any evidence-based or general health information. While 20% (4/20) of the included apps had clinical integration features such as clinician dashboards, only 10% (2/20) had the potential to effectively enhance clinician workflows. Privacy policies were present in 95% (19/20) of the apps, with 75% (15/20) adhering to General Data Protection Regulation (GDPR) regulations; 1 app had no data protection policy. Only 20% (4/20) of the apps were medically certified. For wearables, only 9% (2/22) were tailored to the DACH region, and 40% (9/22) addressed women’s health. While around 60% (13/22) offered features to support clinical integration, only 9% (2/22) had the potential to improve clinical workflows. More than half (12/22) of the wearables were medically certified, and 77% (17/22) referenced scientific or peer-reviewed research. All wearables included a privacy policy.

**Conclusions:**

While many mHealth tools for cardiovascular health are available, only a few provide meaningful value to both patients and clinicians or have the potential to integrate effectively into the health care system. Women’s sex-specific needs are often overlooked, and the benefits for clinicians are limited. In addition, mHealth apps largely lack robust evidence, whereas wearables showed comparatively stronger support through evidence-based and medical certification.

## Introduction

### Background

Cardiovascular diseases (CVDs) are the leading global cause of death, responsible for approximately 17.9 million fatalities annually and affecting over 500 million people worldwide [1-3]. The widespread prevalence of CVD highlights the urgent need for effective diagnosis, treatment, management, and preventive measures [3]. A notable gap in cardiovascular health is the tendency of current approaches to CVD prevention, diagnosis, and treatment to overlook the physiological differences between sexes [4]. This has contributed to a lack of awareness regarding women’s cardiovascular risks, resulting in delayed diagnoses and suboptimal care [5].

The advancement of digital technologies presents a significant opportunity to monitor and manage lifestyle factors and health risks, enabling the early detection and prevention of cardiovascular diseases; through innovative tools like telemonitoring, remote patient monitoring, mobile health (mHealth) apps, and wearable health devices, individuals can engage in proactive health management, providing a transformative approach to cardiovascular care [1]. The World Health Organization’s Global Observatory of Electronic Health (eHealth) considers mHealth a subcategory of eHealth and defines it as “medical and public health practice supported by mobile devices, such as mobile phones, patient monitoring devices, Personal Digital Assistants (PDAs), and other wireless devices” [6]. The integration and widespread adoption of these technologies present a promising shift toward more personalized and proactive health care solutions, with the potential to enhance patient outcomes while simultaneously reducing health care costs [7].

Coorey et al [8] found that mHealth apps significantly reduce hospital readmission rates, improve blood pressure, support healthy dietary habits, and enhance cardiovascular disease management. Smartphones equipped with photoplethysmography technology can detect atrial fibrillation and assess cardiovascular health by measuring blood volume changes using infrared light, this technique provides insights into heart rate and variability, offering a cost-effective and noninvasive method for evaluating cardiovascular fitness [9].

Wearable devices, typically worn on the wrist, arms, chest, or hips, can also be effective tools for managing CVD risks [10]. These devices track various health metrics including heart rate, heart rate variability (HRV), blood oxygen levels, sleep patterns, and physical activity using photoplethysmography or electrocardiogram (ECG) technology to detect atrial fibrillation (AFib) [10,11]. For example, a study by Guo et al [12] involving over 187,000 users identified 265,139 potential AFib cases among 424 users using the smartwatch. Follow-up testing confirmed AFib in 227 out of 262 users, demonstrating that wearables can effectively alert users to potential AFib, prompting timely medical evaluation and early diagnosis. A systematic review of interventions using smartwatches revealed favorable outcomes across various health aspects including improvements in lifestyle changes, medication adherence, reduction in unplanned hospital readmissions, enhanced AFib diagnosis, and better adherence to self-monitoring practices [13].

In the DACH region (Germany, Austria, and Switzerland), cardiovascular diseases also rank as the top cause of death [14]. This region accounts for about one third of Europe’s medical technology market, presenting a key opportunity for health care innovation and research [15]. With its growing focus on health tech, the DACH region is positioned as an emerging leader in advancing medical solutions, making it a compelling market for cardiovascular health interventions [14,15].

### Objectives

In this review, we set out to investigate the landscape of mHealth apps and wearables designed for cardiovascular health globally, with a special focus on tools available in the DACH region. The objective was to identify potential gaps by assessing the value these digital health tools bring to both patients and clinicians. Given the well-documented gap in cardiovascular care related to women’s health, we also aimed to explore whether the analyzed apps and wearables provide tailored support for sub-groups of users, such as addressing sex-specific symptoms. In addition, we aimed to assess the potential of these technologies to integrate into the broader health care ecosystem, with the goal of identifying areas that require improvement and opportunities for future innovation.

## Methods

### Search Strategy

The search for heart health apps involved a screening of the Swiss Apple’s App Store and Google’s Play Store. The Apple Store search was conducted using an iPhone, while Google Play Store apps were identified through its web-based platform. In addition, a supplementary Google search was performed using a combination of English and German keywords to ensure a comprehensive discovery of heart health apps. Keywords were: “heart,” “health,” “heart health,” “cardiac,” “pulse monitor,” “heart rate,” “heart monitor,” “best heart apps,” “fitness app,” “Herz-Apps,” “Herzmonitor,” and “Herzfrequenz.” The search process spanned from April 8, 2024, to June 20, 2024, during which time each app was downloaded, reviewed, and evaluated.

The screening for wearables took place via Google search aimed at identifying the most relevant heart health wearables available both in the DACH region and globally. The keywords “heart wearables,” “heart device tracking,” “health wearables,” “healthcare wearable devices,” “smart wearables,” “fitness wearables,” “heart health tracking,” “medical trackers,” “DACH herzmonitor,” and “Herzmonitor” were used. The search for wearables occurred between April 8, 2024, and July 11, 2024, during which time each vendor’s website and accompanying app if available were thoroughly reviewed, and evaluated.

### Inclusion and Exclusion Criteria

The included apps were required to meet one of two criteria: (1) measure or log health and well-being, typically categorized as health and fitness apps, (2) measure specific metrics such as heart rate and heart rate variability. Apps that did not meet at least one of these criteria were excluded.

Similarly, the selection of wearables was based on two criteria: (1) focus on health and wellness features, (2) possess the capability to measure health vitals such as heart rate, heart rate variability, or ECG. Only wearables meeting both criteria were included. The inclusion criteria of the mobile apps and wearables included in this review are outlined in Textbox 1.

Textbox 1. **Inclusion criteria of the identified mobile apps and wearable devices**

The included mobile apps were required to meet at least 1 of the following criteria:

- Health and well-being tracking: apps that measured or logged health and well-being were typically categorized as health and fitness apps.
- Specific metric measurement: apps that measured specific metrics, such as heart rate and heart rate variability.

The included wearable devices were required to meet both of the following criteria:

- Health and wellness features: wearables offering features related to health and wellness.
- Health vitals measurement: wearables had to be capable of measuring key health vitals, such as heart rate, heart rate variability, or ECG.

### Analysis of Included Apps and Wearables

Each app that met the criteria was downloaded, analyzed, and evaluated. In addition, each website associated with the identified wearables, as well as the accompanying apps where available, were reviewed and downloaded. The features detailed on the wearable companies’ websites were then used to assess their core functionalities and offerings. The first author (GC) devoted 2‐3 hours to testing and evaluating each included app and wearable. The assessments were then reviewed by the last author (CJ), and any discrepancies or disagreements were collaboratively discussed and resolved between them.

An app or wearable was deemed DACH-specific based on 2 key criteria. Firstly, it had to be explicitly designed for the German-speaking region and the DACH countries. While simply providing an app or wearable in German did not automatically qualify it as DACH-specific, those developed by creators from Germany, Austria, or Switzerland, with a focus on their respective health care systems, were deemed as such. Secondly, the app or wearable was considered DACH-specific if the company behind it was based in one of the DACH countries.

The analysis was guided by a sociotechnical framework for evaluating patient-facing eHealth tools, which highlights the significance of context in assessing health care technologies [16]. This framework considers critical factors affecting the adoption of mHealth solutions by both patients and clinicians and evaluates how these technologies fit within the broader health care ecosystem [16-20]. To determine the value for patients, the evaluation focused on the technical features, safety, and functionality of each app and wearable from the patient’s perspective. For clinicians, the analysis extended to include a review of the potential impact on clinical workflow, clinical utility, and the presence of a cost-benefit analysis to substantiate cost-efficiency claims. In addition, the evaluation criteria encompassed data protection, safety and regulatory compliance, interoperability and data sharing, revenue models, and certifications, to assess each tool’s integration within the health care ecosystem. The assessment criteria for the mobile apps and wearables included in this review are outlined in Textbox 2.

Textbox 2. **Assessment criteria for the included mobile apps and wearable devices.**

**General features**

- Do they offer features relevant for CVD such as heart rate measurements?
- Are they DACH (Germany, Austria, and Switzerland) specific?
- Do they offer tailored support to underserved subgroups of users such as sex-specific symptoms?

**Value for patients**

- What are the main benefits for patients?
- Did they consider the whole patient journey? (eg, context, overall treatment path)
- Do they offer proper training and support material for patients?
- Do they provide valid clinical evidence to support their claims? (eg, published results of clinical trials, user research, real world evidence)

**Value for clinicians**

- What are the main benefits for clinicians? And for clinic or hospital management?
- Do they address the potential impact on clinical workflow?
- Do they offer proper training and support material for clinicians?
- Do they provide valid evidence to support their cost-efficiency claims? (eg, a cost-benefit analysis)

**Fit into the ecosystem**

- What is their business model? (eg, are they transparent about how they get paid and by whom?)
- Do they explicitly provide information about their data management and privacy policies?
- Do they address data sharing and interoperability?
- Does the tool require any additional infrastructure to function?
- Are they properly certified according to their risk tier? (eg, FDA [US Food and Drug Administration] approved, CE [Conformité Européenne] marked, certified as software as a medical device)

## Results

### Search Results

**Figure 1.**
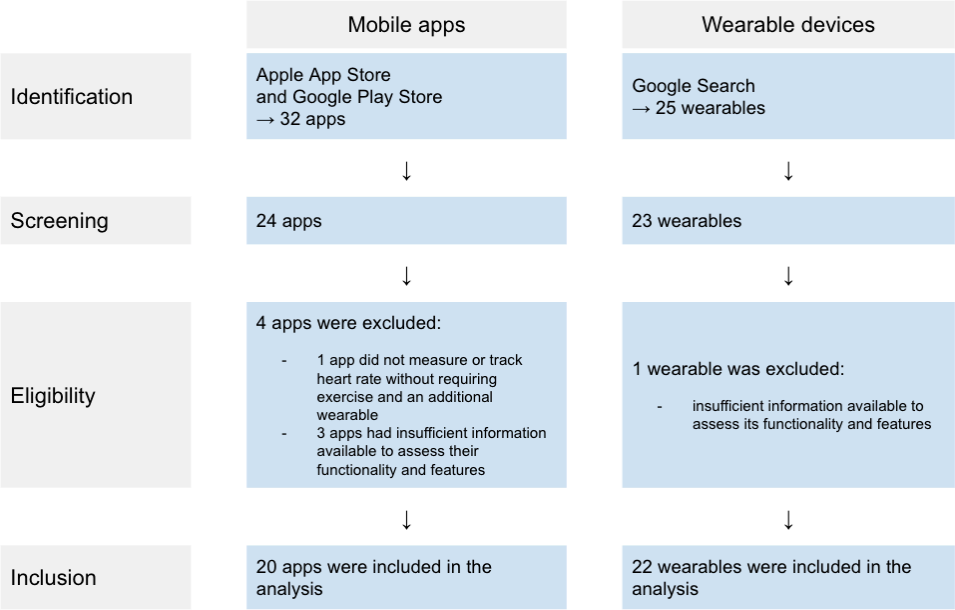
Apps and wearables selection diagram.

Only 20 out of 24 identified apps were included in the analysis. The 4 excluded apps were removed for the following reasons: one app did not measure or track heart rate without requiring exercise and an additional wearable; the remaining 3 apps had insufficient information available to assess their functionality and features. Out of 23 identified wearables, 1 wearable was excluded due to insufficient information available to assess its functionality and features, despite being intended for patient use. The selection diagram for apps and wearables is illustrated in Figure 1.

### Mobile Apps for Managing Cardiovascular Health

Ultimately, 20 apps were included according to the inclusion criteria. Only 30% (6/20) of the apps are tailored specifically for the DACH region, with just one, Preventicus Heartbeats GmbH, providing comprehensive heart health monitoring by measuring vital metrics, including heart rate, and integrating clinical research. This app is Conformité Européenne (CE)–certified and focuses on delivering valuable insights into heart health, particularly for women, which is not commonly addressed by other apps.

Our analysis highlighted that apps can serve either as heart rate measurement tools using photoplethysmography technology or as logbooks or tracking systems. Around 60% (12/20) of apps use the phone’s photoplethysmography capabilities, offering a convenient way to measure heart rate without additional devices. Conversely, apps like Heart Analyzer (Helix Apps Ltd) and Cardiogram (Cardiogram Inc), which do not use photoplethysmography, rely on external devices for data collection. Furthermore, some apps with photoplethysmography functionality, such as Cardi Mate (Gismart), require users to watch advertisements before taking a measurement.

Regarding sex-specific considerations, only 25% (5/20) of the apps address women’s specific health needs. Preventicus Heartbeats GmbH is notable for explicitly including women in its analysis, particularly menopausal women, and assessing the impact of hormonal changes on heart health. Other apps like Welltory (Welltory Inc) and Azumio’s InPulse heart rate monitor include sex-specific considerations but do not provide as detailed an analysis.

Out of the reviewed apps, only 40% (8/20) are supported by scientific research to validate their claims. Meanwhile, 35% (7/20) provide general heart health information but lack direct research support. And 25% (5/20) include neither scientific evidence nor general health data, raising concerns about their credibility.

In terms of value for health care providers, 60% (12/20) of the apps allow users to share heart health reports with clinicians, which enhances their practical value in clinical settings. However, only 20% (4/20) offer platforms for clinicians to manage patient data or provide telehealth services, and just 10% (2/20) positively impact clinician workflows by facilitating appointment bookings, prescription management, or offering interoperability with patient health records.

Most apps (60%, 12/20) are interoperable with devices like Bluetooth-enabled blood pressure monitors or wearables. However, the ability to export and share data from the app itself is available in 75% (15/20) of the apps. The predominant revenue model is a free download with in-app purchases, used by 70% (14/20) of the apps. Only one app, ProHerz (ProCarement GmbH), is offering insurance coverage in Germany with a doctor’s prescription.

Privacy is comprehensively addressed, with 95% (19/20) of apps including a privacy policy and 75% (15/20) ensuring General Data Protection Regulation (GDPR) compliance. Only 20% lack explicit GDPR mention but still provide privacy policies. Finally, 20% (4/20) of the apps are medically certified, with 3, Preventicus Heartbeats GmbH, CardioSignal, and FibriCheck, being clinically tested and capable of detecting AFib. Multimedia Appendix 1 provides a detailed analysis of the included apps based on predefined assessment criteria.

**Figure 2.**
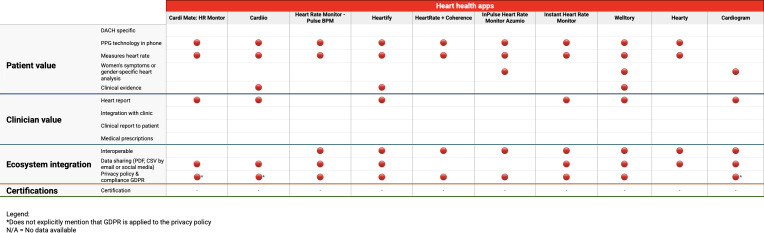
Analysis of each of the included apps – part I. BPM: beats per minute; DACH: Germany, Austria, and Switzerland; GDPR: General Data Protection Regulation; HR: heart rate; PPG: photoplethysmography.

Table 1 provides a consolidated overview of the characteristics of the analyzed apps. Figures2  3 offer a detailed overview of the assessment of each included app, categorized into two distinct groups: “Heart Health Apps” and “Certified Apps.” The Heart Health Apps are distinguished by a red color coding, indicating that these apps either use photoplethysmography technology to measure heart rate directly through the smartphone or support data logging from other devices such as Bluetooth sensors or blood pressure monitors. These apps mostly function as trackers or logbooks for recording heart health metrics. Conversely, certified apps are highlighted in green, signifying that these apps possess formal certifications and evidence-based information. This includes various certifications such as the CE mark or FDA (US Food and Drug Administration) approval.

**Table 1.** Aggregated analysis of the included apps.

App characteristics	Value (N=20), n (%)
DACH^a^ specific	6 (30)
Value for patients	
Measure heart rate	12 (60)
Medical diagnosis	3 (15)
Training and support	12 (60)
Women-specific measures	5 (25)
Value for clinician	
Receives heart report	12 (60)
Clinician integration	4 (20)
Positive impact on workflow	2 (10)
Training and support	3 (15)
Fit into the ecosystem	
Interoperable	12 (60)
Data sharing	15 (75)
Revenue model type	
Free download and no in-app purchase	5 (25)
Free download and with in-app purchase	14 (70)
Insurance covered	1 (5)
Evidence-based	
Research highlighted	8 (40)
General knowledge, no references	7 (35)
No research or scientific evidence	5 (25)
Privacy policy	
GDPR-^b^ or FADP^c^-compliant	15 (75)
Privacy policy but not GDPR-compliant	4 (20)
No privacy policy	1 (5)
Certification	
Certified as a medical app	4 (20)
Not certified	16 (80)

a DACH: Germany, Austria, and Switzerland.

b GDPR: General Data Protection Regulation.

c FADP: Swiss Federal Act on Data Protection.

**Figure 3.**
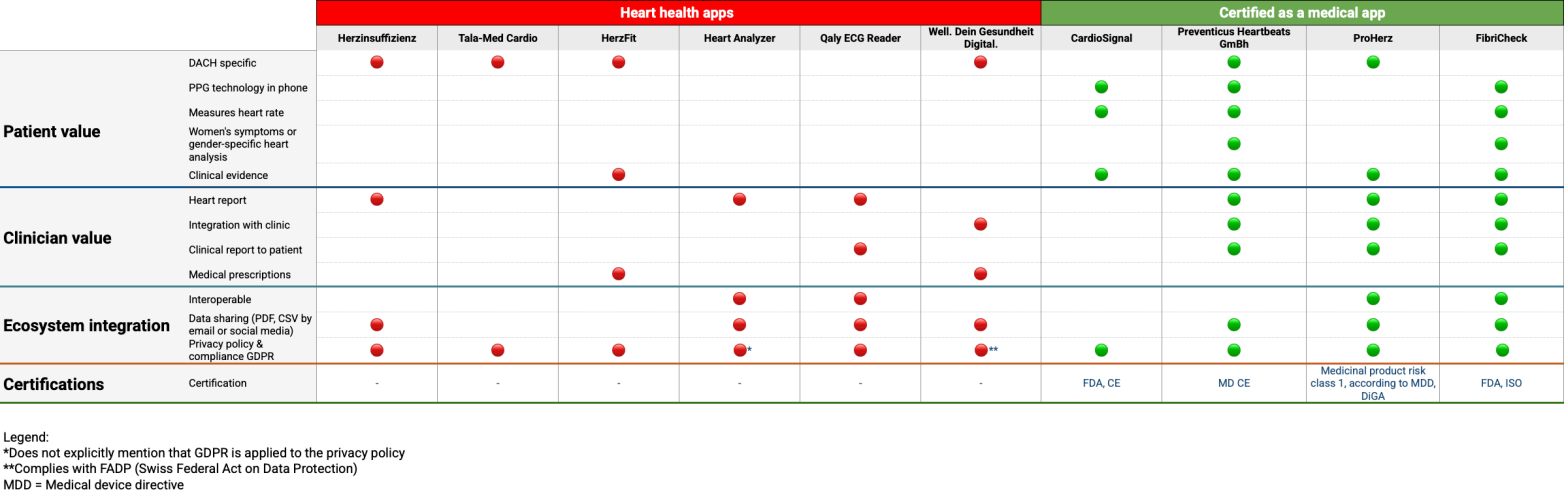
Analysis of each of the included apps – part II. CE: Conformité Européenne; DACH: Germany, Austria, and Switzerland; DiGA: digital health applications; ECG: electrocardiogram; FDA: US Food and Drug Administration; GDPR: General Data Protection Regulation; HR: heart rate; ISO: International Organization for Standardization; PPG: photoplethysmography.

### Wearable Devices for Managing Cardiovascular Health

The analysis of wearables reveals that only 9% (2/22) are specific to the DACH region, with Aktiia (Aktiia SA) and SmartCardia (SmartCardia SA) representing Swiss companies focused on cardiovascular health. Aktiia emphasizes blood pressure measurement, while SmartCardia specializes in detecting AFib.

Among the wearables, all measure heart rate, but only 55% (12/22) provide reliable measures through medically certified devices. Photoplethysmography technology is used by 9 wearables, whereas 13 use ECG for detecting heart irregularities. Most wearables (82%, 18/22) offer comprehensive user instructions. However, only 41% (9/22) explicitly include features for women, such as menstrual cycle tracking or sex-specific heart rate analysis.

The Oura Ring (Oura Health Ltd) and Whoop (Whoop Inc) are particularly notable for their benefits to women. The Oura Ring tracks menstrual cycles and body temperature fluctuations, providing insights into heart health changes throughout the cycle. Whoop offers personalized pregnancy insights and tracks various health metrics through a daily journal.

From a clinical standpoint, wearables can be categorized into 3 distinct groups: fitness-centric, quality-certified, and medically certified. Fitness-centric wearables are primarily designed for general health and fitness purposes, emphasizing exercise and recovery rather than detailed heart health metrics. In contrast, quality-certified and medically certified wearables provide more comprehensive heart health reports, offering valuable data for clinical evaluation. However, a notable gap exists in the cost-benefit analysis provided by these wearables. Most lack robust evidence supporting their efficiency claims, with Philips Mobile Cardiac being the exception. Philips Mobile Cardiac stands out by presenting research that highlights its cost-effectiveness compared to alternative monitoring methods.

All of the included wearables had a privacy policy in place. Among them, 77% (17/22) were compliant with GDPR or Swiss Federal Act on Data Protection (FADP) standards, while 23% (5/22) included a privacy policy but did not explicitly mention GDPR compliance. While integration with clinical workflows varied: Fitness-centric wearables do not support clinician integration, some quality certified wearables offer APIs for data sharing, and medically certified wearables often have platforms for real-time remote monitoring. All wearables support data sharing with smartphones and allow the export of data, though medically certified devices often provide automatic synchronization.

Revenue models vary, with 50% (11/22) of wearables requiring a one-time purchase and 32% (7/22) using a subscription model. Over 75% (17/22) are backed by research or clinical trials, while the rest provide minimal or no scientific evidence. Privacy policies are universally provided, with most wearables ensuring GDPR or FADP compliance. About two-thirds of the wearables have certifications, with 32% (7/22) being CE or ISO (International Organization for Standardization)–certified and 55% (12/24) holding medical certifications such as CE marks or FDA approvals. The distinction between certified and medically certified wearables often lies in the explicit proof of certification and medical approval. Multimedia Appendix 2 provides a detailed analysis of the included wearables based on the predefined assessment criteria.

**Figure 4.**
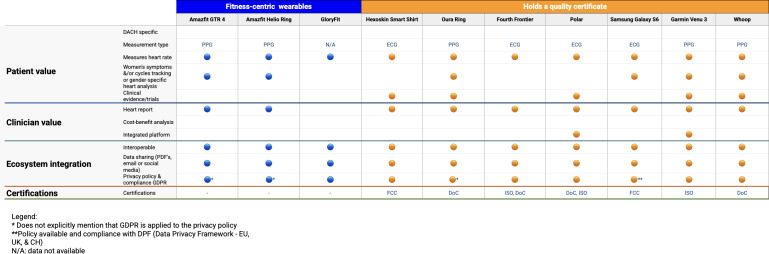
Analysis of each of the included wearables – part I. DACH: Germany, Austria, and Switzerland; DoC: Declaration of Conformity; ECG: electrocardiogram; FCC: Federal Communications Commission; GDPR: General Data Protection Regulation; ISO: International Organization for Standardization; PPG: photoplethysmography.

Table 2 provides a consolidated overview of the characteristics of the analyzed wearables. Figures4  5 offer a detailed overview of the assessment of each included wearable, categorized into 3 distinct groups: “Fitness-centric wearables,” “Quality certified wearables,” and “Medically certified wearables”. Fitness-centric wearables are represented in blue, indicating their primary focus on general fitness and training. Wearables with quality certification, marked in orange, offer detailed health measurements, including heart rate, blood oxygen levels, stress, and recovery, while also meeting quality certification standards such as FCC (Federal Communications Commission), DoC (Declaration of Conformity), or ISO. Wearables certified as medical devices are distinguished in purple, denoting their status as medical devices with regulatory clearance such as FDA clearance, or CE mark, enabling their use in clinical settings and providing a higher level of accuracy.

**Table 2.** Aggregated analysis of the included wearables.

Wearables characteristics	Value (N=22), n (%)
DACH^a^ specific	2 (9)
Value for patients	
Measure heart rate	22 (100)
Medical diagnosis	12 (55)
Training and support	18 (82)
Women-specific measures	9 (41)
Value for clinician	
Receives heart report	18 (82)
Clinician integration	13 (59)
Positive impact on workflow	2 (9)
Training and support	9 (41)
Fit into the ecosystem	
Interoperable	22 (100)
Data sharing	22 (100)
Revenue model type	
One-off purchase of the wearable	11 (50)
Subscription-based app with wearable	7 (32)
Not stated	4 (18)
Evidence-based	
Research highlighted	17 (77)
General knowledge, no references	4 (18)
No research or scientific evidence	1 (5)
Privacy policy	
GDPR-^b^ or FADP^c^-compliant	17 (77)
Privacy policy but not GDPR-compliant	5 (23)
No privacy policy	0 (0)
Certification	
Holds a quality certificate	7 (32)
Certified as a medical device	12 (55)
Not certified	3 (14)

a DACH: Germany, Austria, and Switzerland.

b GDPR: General Data Protection Regulation.

c FADP: Swiss Federal Act on Data Protection.

**Figure 5.**
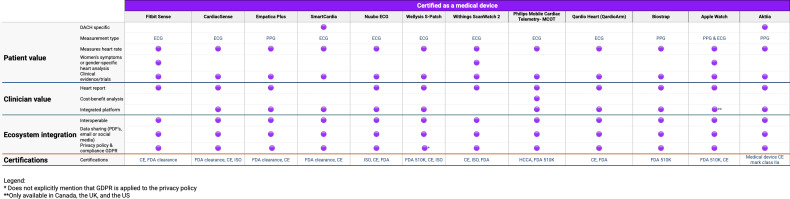
Analysis of each of the included wearables – part II. CE: Conformité Européenne; DACH: Germany, Austria, and Switzerland; ECG: electrocardiogram; FDA: US Food and Drug Administration; GDPR: General Data Protection Regulation; HCCA: Health Care Compliance Association; ISO: International Organization for Standardization; PPG: photoplethysmography.

## Discussion

### Primary Findings

Our analysis showed that many apps do not provide a solid evidence base to substantiate their claims, and many apps lack medical certification and scientific validation, which may diminish trust and adoption by both users and clinicians. Conversely, wearables showed greater credibility, with many devices supported by evidence-based claims and validated through medical certifications. Privacy concerns also emerged as some apps fail to provide clear GDPR-compliant privacy policies, raising risks around data security and user trust. Usability remains a challenge, with some apps requiring additional devices for tracking, which can discourage engagement. Clinical integration faces significant barriers, including few clinician-specific features such as dashboards, and little information about interoperability and data sharing possibilities, limiting the effective integration of these tools in clinical practice. Furthermore, reimbursement remains a major hurdle, as most of the included apps and wearables rely on user payments with only 1 app that is insurance-covered.

### Value for Patients

Our analysis highlights a significant presence of mHealth apps for managing heart health available in the DACH region. However, only 30% (6/20) of the apps are specifically tailored for this region, and just 20% (4/20) are medically certified. Only 40% (8/20) of the apps incorporate scientific research to validate their claims, while 35% (7/20) of the apps provide general heart health information without specific research backing. It is worth noting that 25% (5/20) of the apps lack any scientific or general health data, underscoring a notable gap in clinical research and evidence supporting these apps. This can prove problematic as the absence of clinical trials and scientific validation can undermine both user trust and clinician endorsement. The credibility of mHealth apps relies heavily on accessible, cited scientific research [21]. Without such evidence, users may question the app’s reliability, and clinicians may hesitate to recommend these tools, impacting their adoption in health care [21]. Wearables, on the other hand, performed notably better in this area, with over 75% (17/22) supported by research or clinical trials, providing a stronger evidence base compared to the included apps.

From a usability perspective, our review indicates that 40% (8/20) of the analyzed apps do not enable users to measure their heart rate directly using their smartphones and instead require users to pair the app with an additional device, such as a wearable or a blood pressure monitor, to log and track their heart health. This additional requirement can be cumbersome and may discourage users from engaging with the app, as highlighted by research showing that manual tracking methods often reduce user readiness for self-monitoring [22]. Some apps in our analysis use the index finger for AFib measurements, others require users to lie down and place the phone on their sternum, which may not be practical in all situations, particularly if a rapid heart rate change occurs. Wearables offer a distinct advantage by allowing heart vitals to be measured directly and automatically synced with a mobile app. Previous research showed that users prefer devices like Fitbit trackers for their seamless integration and automatic data synchronization [22]. This automatic syncing not only benefits users but also provides clinicians with valuable insights into the patient’s activities, such as exercise intensity and duration, which are essential for accurate monitoring and assessment [22].

### Value for Clinicians

Over half of the reviewed apps and wearables offer some level of clinician integration, including features like dashboards for remote patient monitoring and data reporting. However, many fall short of significantly improving clinicians’ workflows. Key functionalities, such as appointment scheduling and e-prescription capabilities, are often missing, and important aspects like interoperability with electronic health record are not adequately addressed in the information provided on their websites and supporting documentation. This disconnect complicates processes for both patients and health care providers. Many clinicians are cautious about these tools due to their poor interoperability with existing health care systems and electronic health records, which limits their impact on workflow efficiency [19,20]. Furthermore, research indicates that patients are more likely to trust tools that are recommended by a physician [18,23]; this underscores the necessity of involving clinicians in the app’s use to build trust and enhance its effectiveness.

The lack of scientific evidence, particularly among the identified apps (with wearables performing slightly better in this regard), may pose a challenge for clinician adoption. Previous research has shown that clinicians are often hesitant to recommend mHealth apps due to concerns about insufficient evidence and supporting research. A survey found that 62% of physiotherapists would not recommend apps lacking sufficient evidence and quality due to potential risks to patient health [24]. Similarly, 81.1% of general practitioners believe that mHealth apps should undergo clinical testing and receive certification from independent experts to ensure their effectiveness and reliability [25].

Our analysis suggests that medically certified apps and wearables are better-suited to support clinical integration, as the evidence base required for certification may enhance their credibility and acceptance among medical professionals. However, their effectiveness can still be hindered by several barriers. Achieving seamless integration into clinicians’ workflows is vital to enable efficient remote monitoring and promote effective 2-way communication between patients and health care providers. Furthermore, challenges such as lack of compensation, increased workload, and insufficient digital literacy are significant obstacles for clinician adoption [19,20].

### Fit Into the Ecosystem

According to the analysis, 75% of the reviewed apps explicitly state their GDPR compliance, while 20% do not mention it at all, and 1 app, the Hearty app (Pathfinder DMCC), lacks a privacy policy altogether. All of the included wearables featured a privacy policy. Of these, 77% (17/22) adhered to GDPR or FADP standards, ensuring compliance with data protection regulations. However, 23% (5/22) had a privacy policy in place but made no explicit reference to GDPR compliance, leaving room for ambiguity regarding their adherence to these standards. Previous research demonstrated the crucial role of privacy policies and data protection in building user trust, indicating that apps with privacy policies adhering to GDPR and employing high-security measures are viewed as more trustworthy [21].

GDPR regulations require that privacy policies be prominently displayed on an app’s landing page and remain accessible throughout use [26]. However, some heart health apps analyzed in this review fall short of these transparency standards, making it harder for users to access their privacy policies. Noncompliance with GDPR can erode user trust, as mHealth apps often handle sensitive personal data shared with clinicians, family members, or third parties [21]. This kind of noncompliance poses risks like data breaches and unauthorized access to personal information, which can adversely affect user perceptions [27]. Apps with unclear or insecure privacy policies may face adoption barriers [28].

Our analysis highlights a significant gap in demonstrating cost reimbursement for eHealth tools. Among the included apps, only one is covered by health insurance, while the majority rely on in-app purchases for funding. Wearables, on the other hand, are generally available either as one-time purchases or through subscription-based models. Despite evidence suggesting that mHealth wearables can reduce stroke frequency and lower costs per stroke for high-risk patients, the lack of a clear reimbursement model remains a major barrier [29]. Previous research indicates that while initial costs for using wearables to detect AFib may be higher, they ultimately lead to reduced stroke incidence and lower overall costs due to effective anticoagulation therapy [29].

Another significant gap lies in the cost-benefit analyses provided by the included apps and wearables, as most fail to offer robust evidence to substantiate their cost-efficiency claims. An exception is Philips Mobile Cardiac, which distinguishes itself by presenting research that demonstrates its cost-effectiveness when compared to other monitoring methods. This adds to the challenges faced by health care providers in integrating mHealth tools primarily due to reimbursement issues [30]. The complex reimbursement pathways and lack of incentives for remote care significantly hinder the adoption of these technologies, particularly when their cost-efficiency claims are not supported by robust evidence [31].

### Limitations and Future Research

In our review, we faced several limitations that should be acknowledged. The rapid evolution of technology means that the apps and wearables included in our assessment may have undergone updates or feature changes since the research was conducted, potentially altering their functionalities. In addition, some apps and wearables were inaccessible to us due to stringent verification requirements, such as restrictions limiting access to paying users only. While we evaluated whether the apps and wearables included statements about data security, we were unable to verify whether these tools effectively implemented secure data-handling practices. Moreover, despite our efforts to use comprehensive search terms to capture as many relevant apps and wearables as possible, there remains a possibility that some significant tools were inadvertently excluded.

It is also important to note that our quality assessment was conducted as a high-level narrative review, focusing primarily on the features we could test after downloading the apps and information provided by developers through their websites and related documentation. This approach did not extend to verifying the success of real-world implementation, evaluating the quality of specific claimed functionalities (eg, integration into clinical workflows or interoperability), or assessing long-term performance in practical health care settings. Future studies could complement our initial findings by conducting comprehensive evaluations under real-world conditions. Such research would enable a deeper understanding of these tools’ effectiveness, including their performance over extended periods and their impact on specific health care workflows, offering a more detailed comparison of their overall quality and value.

### Conclusion

Our findings showed that mHealth apps and wearables hold considerable promise for improving the interaction between patients and clinicians in the management of cardiovascular diseases. We identified several strengths in these technologies, including accurate measurements, robust privacy policies, and useful clinical features. However, our analysis showed that the mHealth apps included in the study largely lack a solid foundation of scientific evidence. In contrast, wearables demonstrated a higher degree of credibility, with many supported by evidence-based claims and validated through medical certification.

Significant gaps remain, particularly in addressing women’s cardiovascular health. A notable percentage of apps and wearables do not adequately meet the specific needs of women, who experience cardiovascular diseases differently than men. This underrepresentation in clinical research results in a lack of tailored diagnostic and treatment options, leading to less effective care. Our findings emphasize the need for health apps and wearables that are more inclusive of sex-specific cardiovascular needs throughout different life stages, such as pregnancy and menopause.

In addition, our results show that there is a critical need for better integration of these technologies into clinical practices through features that allow better integration into clinical workflows and interoperability with hospital IT systems. Our assessment indicates that most of the current mHealth tools do not offer enough evidence to improve clinician workflows sufficiently, limiting their ability to provide seamless and efficient care. Further research and case studies are required to demonstrate the financial benefits of these technologies as they mostly lack any evidence to support their cost-effectiveness claims. These gaps present a vital opportunity for innovation in mHealth, fostering stronger connections between patients and clinicians while ultimately improving heart health outcomes for all.

## Supplementary material

10.2196/65782 Multimedia Appendix 1 Analysis of the included apps based on the predefined assessment criteria.

10.2196/65782 Multimedia Appendix 2 Analysis of the included wearables based on the predefined assessment criteria.

## Abbreviations

AFib: atrial fibrillation
CE: Conformité Européenne
CVD: cardiovascular disease
DACH: Germany, Austria, and Switzerland
DoC: Declaration of Conformity
FADP: Swiss Federal Act on Data Protection
FCC: Federal Communications Commission
FDA: US Food and Drug Administration
GDPR: General Data Protection Regulation
HRV: heart rate variability
ISO: International Organization for Standardization
mHealth: mobile health
